# Mechanism and therapeutic implications of pomalidomide-induced immune surface marker upregulation in EBV-positive lymphomas

**DOI:** 10.1038/s41598-023-38156-z

**Published:** 2023-07-18

**Authors:** Hannah K. Jaeger, David A. Davis, Ashwin Nair, Prabha Shrestha, Alexandra Stream, Amulya Yaparla, Robert Yarchoan

**Affiliations:** HIV and AIDS Malignancy Branch, Center for Cancer Research, National Cancer Institute, Building 10, Rm. 6N106, MSC 1868, 10 Center Drive, Bethesda, MD 20892-1868 USA

**Keywords:** Cancer, Cancer therapy, Tumour virus infections

## Abstract

Epstein-Barr virus (EBV) downregulates immune surface markers to avoid immune recognition. Pomalidomide (Pom) was previously shown to increase immune surface marker expression in EBV-infected tumor cells. We explored the mechanism by which Pom leads to these effects in EBV-infected cells. Pom increased B7-2/CD86 mRNA, protein, and surface expression in EBV-infected cells but this was virtually eliminated in EBV-infected cells made resistant to Pom-induced cytostatic effects. This indicates that Pom initiates the upregulation of these markers by interacting with its target, cereblon. Interestingly, Pom increased the proinflammatory cytokines IP-10 and MIP-1∝/β in EBV infected cells, supporting a possible role for the phosphoinositide 3-kinase (PI3K)/AKT pathway in Pom’s effects. Idelalisib, an inhibitor of the delta subunit of PI3 Kinase, blocked AKT-Ser phosphorylation and Pom-induced B7-2 surface expression. PU.1 is a downstream target for AKT that is expressed in EBV-infected cells. Pom treatment led to an increase in PU.1 binding to the B7-2 promoter based on ChIP analysis. Thus, our data indicates Pom acts through cereblon leading to degradation of Ikaros and activation of the PI3K/AKT/PU.1 pathway resulting in upregulation of B7-2 mRNA and protein expression. The increased immune recognition in addition to the increases in proinflammatory cytokines upon Pom treatment suggests Pom may be useful in the treatment of EBV-positive lymphomas.

## Introduction

Pomalidomide (Pom) is a cereblon-binding immunomodulatory drug used for the treatment of multiple myeloma (MM), myelodysplastic syndrome (MDS), and erythema nodosum leprosum. Pom has greater potency and reduced toxicity as compared to its analogs, thalidomide and lenalidomide^1–3^. Pom was recently approved by the United States Food and Drug Administration (FDA) for use in Kaposi sarcoma (KS), which is caused by the gamma herpesvirus Kaposi sarcoma herpesvirus (KSHV), also called human herpesvirus-8^4,5^. KSHV is also the etiologic agent of several other diseases including primary effusion lymphoma (PEL), a form of multicentric Castleman disease (MCD), and KSHV-inflammatory cytokine syndrome (KICS). We have been exploring the effects of Pom on virus-infected cells and its role in affecting immune recognition. To avoid immune recognition by the host cell, viruses have evolved multiple ways to down-regulate important immune surface markers such as MHC-I, ICAM-1, B7-2, etc.^6–12^. MHC-I plays an important role in T-cell recognition; ICAM-1 is a cell-adhesion molecule that helps increase T and NK cell activity; and B7-2 (also called CD86) enhances TCR/CD3-mediated activation of T-cells by binding to its receptor CD28^13^. KSHV encoded genes including K3 and K5 have E3 ubiquitin ligase activity and through this mechanism downregulate the surface expression of these immune proteins. We previously demonstrated that Pom could increase the levels of these surface markers in KSHV-infected PEL cells lines^14^ and later showed that this led to increased T-cell and natural killer (NK) cell recognition of the cells^15^. Interestingly, the increases in B7-2 and ICAM-1 induced by Pom in PEL cell lines were not associated with an increase in their mRNA. Most of the cytotoxic and immunomodulatory effects of pomalidomide and related drugs are attributed to its interaction with cereblon, leading to degradation of Ikaros family zinc finger protein 1 and 3 transcription factors (IKZF-1 and IKZF-3), also called Ikaros and Aiolos, respectively, and to subsequent downstream effects related to the downregulation of interferon regulatory factor-4 (IRF4) and c-Myc^1–3^. A KSHV-infected BCBL-1 cell line made resistant to Pom-induced cytotoxicity by our group had a substantial reduction in the levels of cereblon compared to the parent line and no longer manifested Pom-induced immune surface marker upregulation^15^. These studies provided evidence that Pom initiates its effects on immune surface markers through binding to cereblon.

Epstein Barr virus (EBV) is associated with the development of certain forms of non-Hodgkin lymphoma (NHL). In EBV-associated malignancies, EBV utilizes many different pathways to evade the immune system. Specifically, in Burkitt lymphoma, EBV-encoded poly(A)(-) RNAs (EBER’s) have been shown to incur resistance to apoptotic inducible pathways^16^. Also, the early lytic EBV-encoded gene BDLF3 can increase ubiquitination and downregulation of MHC-I and MHC-II^17^. Moreover, latently expressed EBV membrane protein 2A (LMP2A) can induce downregulation of MHC-I and MICA leading to decreased CD8 + T-cell recognition and natural killer (NK) cell induced apoptosis^18^. Our previous studies showed that in addition to its effects in PEL lines, Pom could also increase expression of B7-2 and ICAM-1 in EBV-infected lymphoma cell lines and lead to increased T-cell activation and NK cell recognition of these lines^19^. 

Here, we explored the mechanism responsible for these effects in EBV-infected lymphoma lines and investigated this in detail using the EBV-infected Daudi line. We also evaluated this mechanism in other Burkitt lymphoma lines. While we suspected that Pom’s mechanism of upregulating surface markers in EBV-infected cells would be the same as that for PEL lines, our observations suggest that there were substantial differences. For instance, in contrast to PEL cell lines, the upregulation of B7-2 in EBV infected cells is at least in part, if not all, due to an increase in its mRNA. We provide evidence that Pom acts on the PI3 kinase (PI3K)/AKT pathway to increase these markers in EBV-infected cells. This pathway leads to increased PU.1 binding to the B7-2 promoter resulting in upregulation of B7-2. This is different from the effects of Pom in KSHV-infected PEL cells, which do not express PU.1^20^. Also unique to EBV infected cells, we found that a specific inhibitor of the delta subunit of PI3K was able to prevent B7-2 upregulation by Pom. These data indicate that Pom upregulates surface markers through distinct pathways in cells infected by these two herpesviruses even though the pathways both appear to require cereblon.

## Results

### Pom-resistant cells have decreased cereblon, decreased surface immune markers, and little or no induction of T-cell activation when exposed to Pom

**Figure 1:**
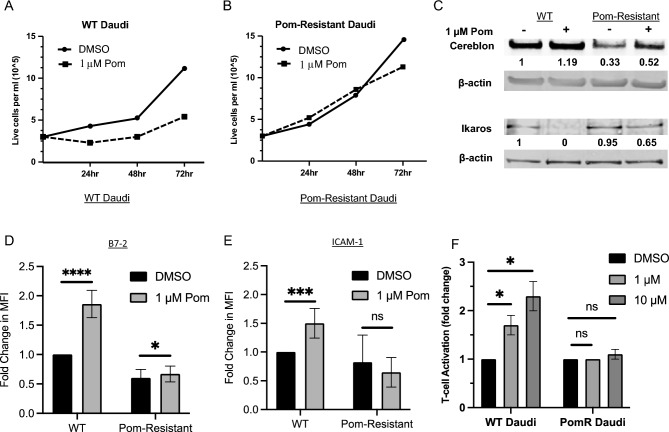
Pom-resistant cells show low cereblon expression and Pom no longer increases surface markers or T-cell activation. Live cell number for (**A**) WT Daudi or (**B**) Pom-Resistant Daudi cells after DMSO (solid) or 1 µM Pom (dashed). (**C**) Representative immunoblots from 3 separate experiments of cytoplasmic extracts for cereblon and nuclear extracts for IKZF-1 following DMSO or 1 µM Pom treatment for 48 h. Protein was normalized to actin and the numbers under the blots represent the fold changes calculated against control (DMSO-treated) cells. (**D**) FACS analysis on live cells showing the mean fold changes + / − SD for B7-2 for DMSO (black bars) and Pom treated (grey bars), compared to DMSO-treated wild type (WT) cells. (**E**) Mean fold changes + / − SD for ICAM-1 for DMSO (black bars) and Pom treated (grey bars). Average fold change in median fluorescent intensity (MFI) compared to DMSO treated cells. Error bars represent standard deviation from 7 independent experiments (****p ≤ 0.0001, ***p ≤ 0.001, *p < 0.05, ns not significant). (**F**) Activation of Jurkat IL-2 reporter cells by WT and Pom-resistant cells exposed to varying amounts of Pom. WT or Pom-resistant Daudi cells were treated with DMSO vehicle, 1 or 10 µM Pom for 2 days and then co-incubated with Jurkat IL-2 reporter T-cells in the presence of varying concentrations of anti-CD3 antibody. Luminescence, a measure of T-cell activation, was measured after 6 h. Viability for BCBL-1 cells was greater than 83% for DMSO, 1 µM and 10 µM Pom. Results show the mean + / − SD from three independent experiments. (*p < 0.05, ns not significant).

We previously demonstrated that Pom increases immune surface markers B7-2 and ICAM-1 in certain EBV infected tumor cell lines and that this leads to increased T-cell activation and NK-mediated cell killing in Daudi cells^19^. To determine if Pom-induced upregulation of these markers was correlated with Pom’s cytostatic effects, we generated an EBV-infected Daudi cell line resistant to Pom’s cytostatic effects by serial passaging with increasing concentrations of Pom up to 8 µM (see ‘Material and Methods’ section for details). The growth of wild type (WT) Daudi cells over a 72 h period was substantially inhibited by 1 µM Pom (Fig. 1A) even though this concentration of Pom did not induce cell death during this time (Supplemental file 2), suggesting Pom was slowing the growth of Daudi cells but not killing them. The growth of the Pom-resistant line, however, was similar to DMSOtreated cells up to 48 h and only showed a minimal (21%) decrease in growth by 72 h (Fig. 1B). Cereblon is the binding target for Pom and has been linked to its cytostatic and anti-myeloma activity^21–23^. Resistance to this anti-myeloma activity has been associated with decreased cereblon expression both in vitro and in clinical trials^24,25^. Consistent with this, our Pom-resistant Daudi cells showed a 67% decrease in the protein levels of cereblon as compared to the respective controls, and the decrease persisted in the presence of 1 µM Pom (Fig. 1C). Although cereblon could still be detected in the resistant line, the 67% decrease was sufficient to prevent most of the Pom-induced cereblon destruction of Ikaros (IKZF1), thus demonstrating a loss of cereblon function (Fig. 1C).

We then investigated the ability of Pom to upregulate B7-2 and ICAM-1 in these two lines. WT Daudi cells showed an average 1.9-fold increase in B7-2 surface expression following Pom treatment, while the resistant line showed only an average 1.12-fold increase when treated with Pom (Fig. 1D). Similarly, surface expression of ICAM-1 increased an average of almost 1.5-fold in WT Daudi cells, while this increase in expression no longer occurred in the resistant line treated with Pom (Fig. 1E). Examples of the surface expression FACS profiles for B7-2 and ICAM-1 and the effect of Pom treatment can be seen in Supplemental file 3. We previously demonstrated that Pom’s effects on immune surface markers are associated with an increase in immune recognition, including increases in T-cell activation 48 h after exposure to Pom-treated Daudi cells^19^. Consistent with these previous results, treatment of WT Daudi cells with 1 and 10 µM Pom for 48 h resulted in enhanced T-cell activation by these cells (Fig. 1F). However, there was no significant increase in T-cell activation in the Pom-resistant Daudi line, consistent with the substantial loss of the upregulation of B7-2 and ICAM-1 (Fig. 1F). Taken together, these data are consistent with Pom acting through cereblon to upregulate the immune surface markers in Daudi cells, resulting in increased immune recognition.

### Pom treatment increases B7-2 mRNA and B7-2 cellular protein levels in Daudi cells

**Figure 2:**
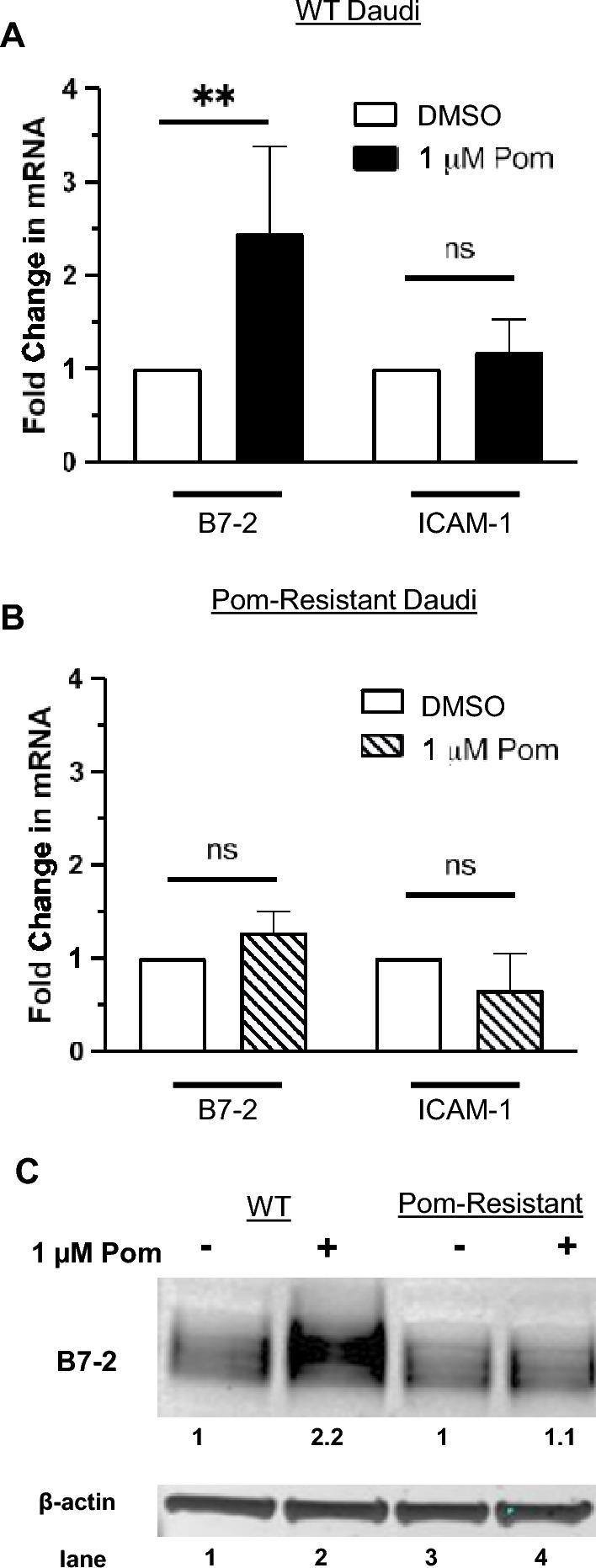
Pom increases the mRNA and protein levels for B7-2 in WT but not in Pom-resistant Daudi cells. (**A**–**B**) Mean mRNA levels of B7-2 and ICAM-1 in (**A**) WT Daudi and (**B**) Pom-Resistant Daudi cells treated with DMSO (control) or Pom 1 µM after 24 h. The mRNA levels of B7-2 and ICAM-1 are normalized to 18S RNA. Error bars represent standard deviation from 7 independent experiments in WT Daudi cells and 3 independent experiments in Pom-Resistant Daudi cells. Statistically significant differences (**p ≤ 0.01, ns not significant) between control and Pom-treated cells are indicated. (**C**) Protein levels by immunoblot of B7-2 and β-actin from cytoplasmic lysates of WT or Pom-Resistant cells 48 h after DMSO or Pom treatment. Fold changes shown under each lane are values normalized to the actin level in the DMSO control for each sample.

To determine if the increases in B7-2 and ICAM-1 were due to increases in their transcription, we assessed mRNA expression levels after treatment of WT Daudi and Pom-resistant Daudi cells with Pom for 24 h. Treatment of WT Daudi cells with 1 μM Pom led to a significant 2.5-fold increase in B7-2 mRNA over its basal expression but interestingly did not induce a significant increase in ICAM-1 mRNA (Fig. 2A). By contrast, B7-2 mRNA expression was not significantly increased in Pom-resistant cells and ICAM-1 mRNA also remained unchanged (Fig. 2B). For B7-2, the change in mRNA was mirrored in changes in the cytoplasmic protein levels of B7-2 with a 2.2-fold increase following Pom treatment of WT Daudi cells but not in Pom-resistant cells (Fig. 2C). We were unable to detect ICAM-1 protein in cellular extracts by immunoblot after trying multiple antibodies. Therefore, we could not assess differences in cellular protein levels for ICAM-1. Overall, these data are consistent with the increase in B7-2 in Daudi cells being caused, at least in part, by an increase in its mRNA although this appears not to be the case for the increase in ICAM-1 surface expression.

### Pom increases the levels of MIP-1∝/β and IP-10 in the supernatant of EBV-infected cells

**Figure 3:**
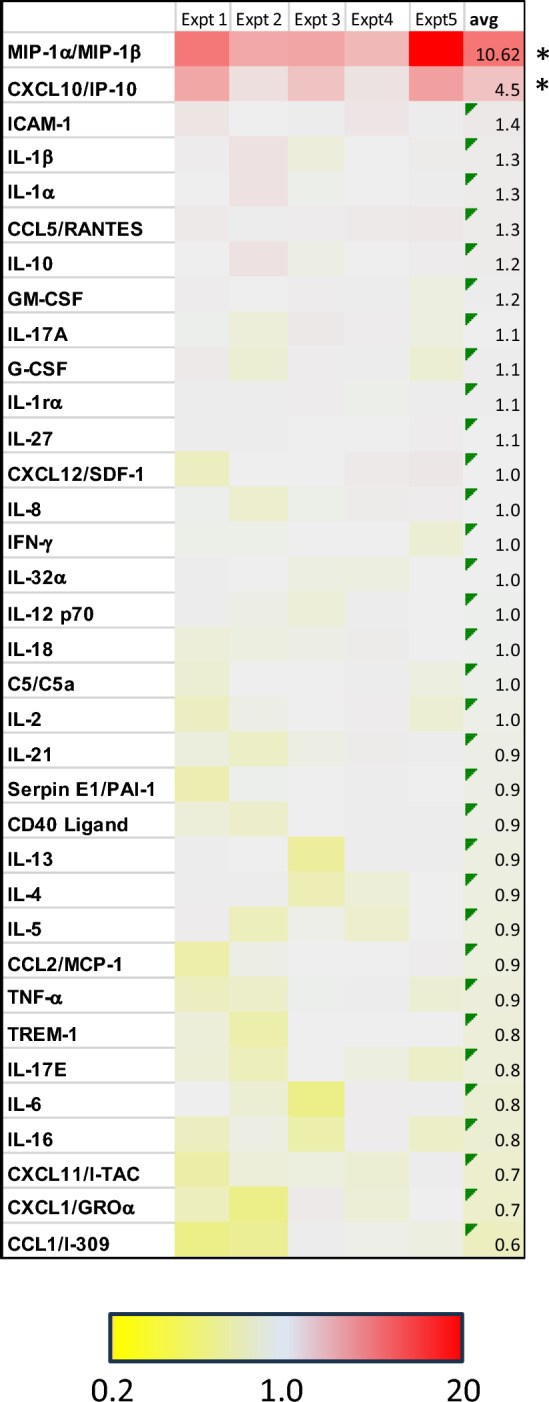
Pom significantly increases MIP-1∝/β and IP-10 in the supernatant of Daudi cells following Pom-treatment. Heat map depicting the fold changes over control for 35 different cytokines. Daudi cells were treated with 1 µM Pom for 48 h and then supernatant was collected and analyzed for changes in cytokine expression using the Proteome Profiler Human Cytokine Array Kit. Shown on the right column are the means for 5 independent experiments run in duplicate. Color range is from 0.2 to 20-fold with yellow indicating a decrease in levels, light blue indicating no change in levels and red indicating an increase in levels. Statistically significant differences between control and Pom-treated cells are indicated for those cytokines whose expression changed by at least an average of twofold (*p ≤ 0.05).

Previous studies have identified several different mechanisms by which B7-2 can be upregulated at the transcriptional level in B-cells and other cell types. Several of these involve cytokines that upregulate B7-2. For example, a combination of IL-1∝, IL-4, IFN-γ and IL-13 was reported to increase B7-2, although this required CD40 receptor stimulation, while IL-21 alone was shown to induce B7-2 transcription in B-cells^26^. Also, it was reported that IL-4 could, by itself, upregulate B7-2 transcription in B-cells^27^. To explore whether Pom may work by upregulating cytokines that mediate the upregulation of B7-2, we assessed Pom’s effect on cytokine secretion in Daudi cells using a cytokine array that measures the levels of over 30 different cytokines. A heat map of the data from 5 separate experiments indicates that IL-1∝, IL-4 and IL-21 levels in the supernatant of Daudi cells treated with 1 µM Pom for 48 h were not significantly affected (less than an average 30% change) (Fig. 3). However, Pom treatment consistently increased both IP-10 and MIP-1∝/β over the control (average 4.5-fold, P < 0.05 for IP-10 and average 10.3-fold, *P* < 0.05 for MIP-1∝/β) (Fig. 3). Of note, the increases in MIP-1∝/β and IP-10 were almost completely abolished in Pom-resistant Daudi cells treated with Pom (Supplemental file 4A,B). We also tested the effect of Pom on other EBV-infected lines as well as KSHV co-infected cells, KSHV-only infected cells and PBMCs. All the EBV-infected lines showed at least a twofold increase in MIP-1∝/β and/or IP-10 while the KSHV-infected line BCBL-1 and PBMC’s showed no increase in either cytokine (Supplemental file 4C,D). While there is evidence that MIP-1∝ activates phosphatidylinositol 3-kinase (PI3K)^28–30^, and this pathway has been implicated in activating PU.1 which is a known to bind and activate the B7-2 promoter, we did not observe an increase in B7-2 or ICAM-1 upon the addition of recombinant MIP-1∝ (Supplemental file 5). As discussed more later, the upregulation of MIP-1∝/β and IP-10 may contribute to tumor responsiveness through additional mechanisms.

### A role for PU.1 and the PI3K/AKT pathway in upregulation of B7-2 surface expression

**Figure 4:**
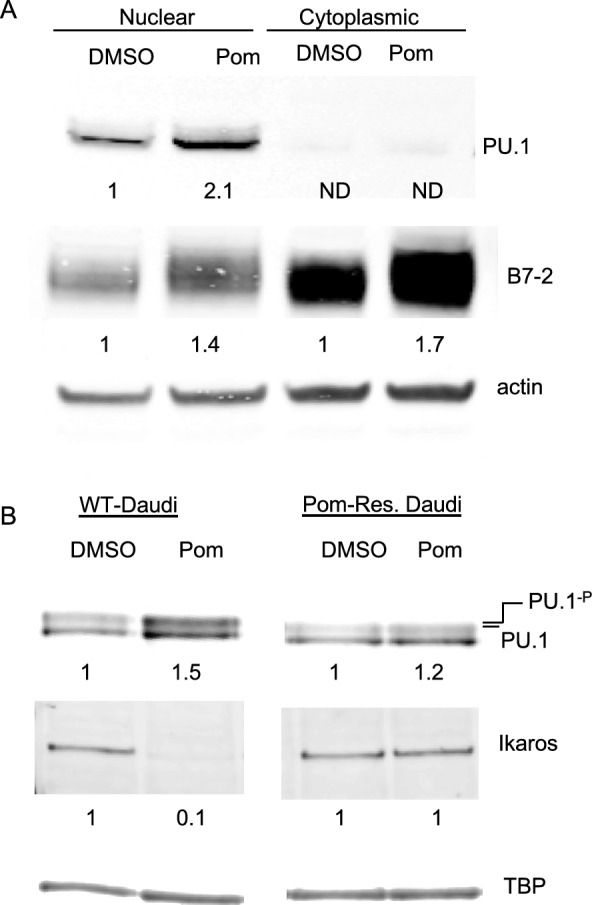
Pom increases PU.1 and B7-2 levels while decreasing Ikaros levels in WT but not in Pom-resistant cells. (**A**) Protein levels by immunoblot (using MES running buffer) for PU.1, B7-2, and β-actin from nuclear and cytoplasmic extracts of WT Daudi cells 48 h after DMSO or 1 µM Pom treatment. Fold changes shown under each lane are values normalized to the actin level in the DMSO control for each extract. (**B**) Protein levels by immunoblot using MOPS running buffer for PU.1, Ikaros, TBP and β-actin from nuclear lysates of WT and Pom-resistant Daudi cells 48 h after DMSO or Pom treatment. Fold changes shown under each lane are values normalized to TBP in the DMSO control for each cell type. Note that PU.1 runs as a doublet when using MOPS running buffer which provides better separation.

We next explored whether PU.1 and the PI3K/AKT pathway might play a role in Pom-induced upregulation of B7-2 mRNA. It is known that treating certain cells with Pom induces cereblon-dependent degradation of Ikaros, which is a repressor of the PI3K pathway^31^. Also, the PI3K/AKT pathway has been shown to be involved in activating PU.1, an E26 transformation-specific transcription factor (ETS)^32^ that directly binds to the B7-2 promoter and can upregulate its mRNA expression in murine and human dendritic cells^29,30^. Pom has also been shown to upregulate PU.1 in myeloma cells^33^. To see if PU.1 might be playing a role in Pom-induced upregulation of B7-2, we first examined the expression of PU.1 in Daudi cells by immunoblot with and without Pom treatment. Pom at 1 µM increased the levels of PU.1 protein in nuclear extracts 2.1-fold (Fig. 4A). This increase in PU.1 coincided with 1.4 and 1.7-fold increases in B7-2 protein in the nuclear and cytoplasmic fractions, respectively, of Pom-treated Daudi cells (Fig. 4A). Interestingly, when protein samples from nuclear extracts were run on gels with the InVitrogen MOPS buffer, we could consistently see PU.1 run as a doublet (Fig. 4B and Supplemental file 6) consistent with the upper band being a phosphorylated form of PU.1, as demonstrated by others^32,34^. Both bands increased in intensity upon Pom treatment in WT Daudi cells but did not substantially change in the Pom-resistant cells (Fig. 4B). Also, the increase in PU.1 due to Pom treatment coincided with a dramatic loss of Ikaros in WT-cells, but this was not seen in Pom-resistant cells (Fig. 4B).

**Figure 5:**
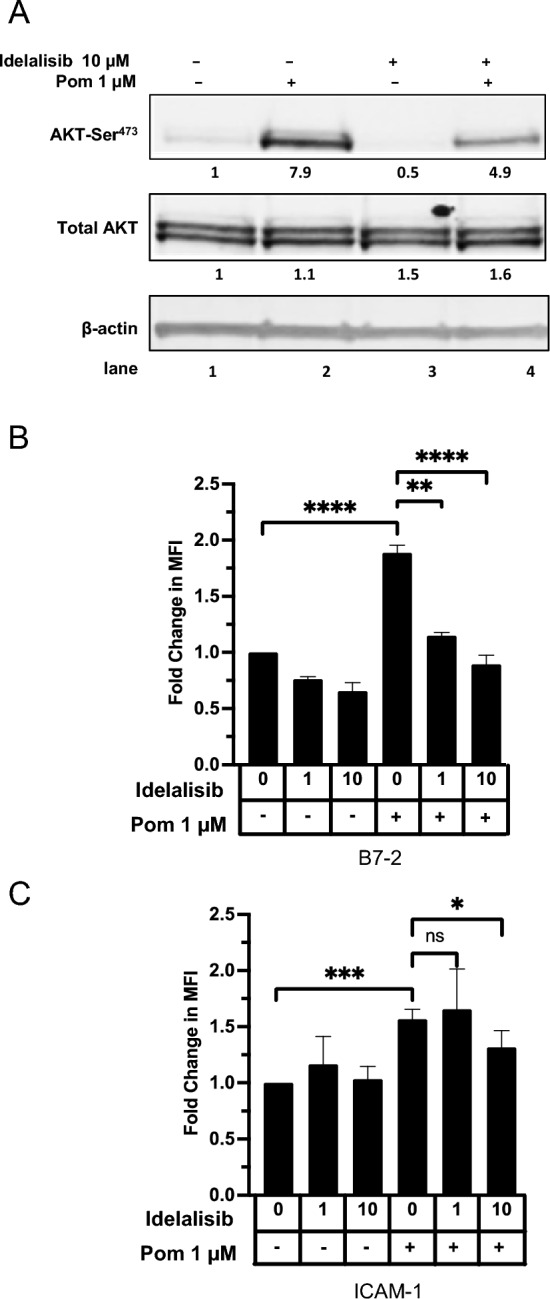
Idelalisib inhibits AKT-Ser^473^ phosphorylation and inhibits B7-2 surface expression induced by Pom. Daudi cells were pretreated with DMSO or PI3K δ-subunit inhibitor idelalisib for 2 h and then treated with 1 μM Pom for an additional 48 h. (**A**) Representative immunoblots of cytoplasmic extracts show AKT-Ser^473^ phosphorylation levels. Fold change of AKT phosphorylation was calculated by first normalizing to β-actin, followed by normalization to total AKT present and then to their respective DMSO controls. (**B**–**C**) Surface expression levels of (**B**) B7-2 and (C) ICAM-1 were measured by flow cytometry using PerCP/Cy5.5-conjugated anti-B7-or anti-ICAM-1 antibodies in Daudi cells treated with Pom (1 μM) and/or idelalisib (1 or 10 μM). Graphs show fold changes in median fluorescence intensity (MFI) of (**B**) B7-2 and (**C**) ICAM-1 upon treatment with Pom and/or idelalisib relative to DMSO-treated cells. Shown are the means ± standard deviations of at least 4 separate experiments. Statistically significant differences (****p ≤ 0.0001, ***p ≤ 0.001, **p ≤ 0.01, *p ≤ 0.05, ns not significant) between control and Pom-treated cells are indicated. The immunoblots were performed 3 times and the result from one representative experiment is shown.

We conducted further experiments to explore a possible role of the PI3K/AKT pathway in activating PU.1. Phosphorylation at serine 41 of PU.1 in B-cells can be carried out by AKT in a PI3K dependent manner, leading to activation of PU.1; this activation is critical for PU.1’s activity in upregulating target genes^35–38^. We hypothesized that Pom might be increasing B7-2 mRNA by activating PI3K, leading to AKT phosphorylation and subsequent phosphorylation of PU.1. Incubation of Daudi cells with 1 µM Pom led to a 7.9-fold increase in phosphorylation of AKT Ser^473^ (Fig. 5A, Lanes 1 and 2). Previous studies have demonstrated that AKT Ser phosphorylation can be inhibited in certain malignant B-cells using the selective inhibitor idelalisib, which blocks activity of the delta subunit of PI3K (PI3Kδ)^39,40^. Therefore, we investigated the ability of idelalisib to inhibit the Pom-induced increases in AKT-ser phosphorylation and subsequent increases in immune surface expression of B7-2 and ICAM-1 in Daudi cells. Idelalisib, at 10 µM, decreased both basal and Pom-induced upregulation of AKT-Ser^473^ phosphorylation in Daudi cells (Fig. 5A, lanes 3 and 4). Importantly, 1 and 10 µm idelalisib also significantly inhibited the Pom-induced upregulation of B7-2 (Fig. 5B) but overall had relatively little effect on ICAM-1 expression (Fig. 5C). Interestingly, idelalisib did not significantly affect the basal or Pom-induced surface expression for B7-2 or ICAM-1 in BCBL-1 cells even at 20 µM (Supplemental file 7) suggesting that the delta subunit of PI3K is not involved in increasing surface expression of B7-2 or ICAM-1 in KSHV-infected cells. These data suggest that Pom activates the delta subunit of PI3K in Daudi cells, leading to activation of AKT which may phosphorylate PU.1 and result in increased B7-2 surface expression.

### Pom-treatment increases the binding/occupancy of PU.1 at the B7-2/CD86 promoter

**Figure 6:**
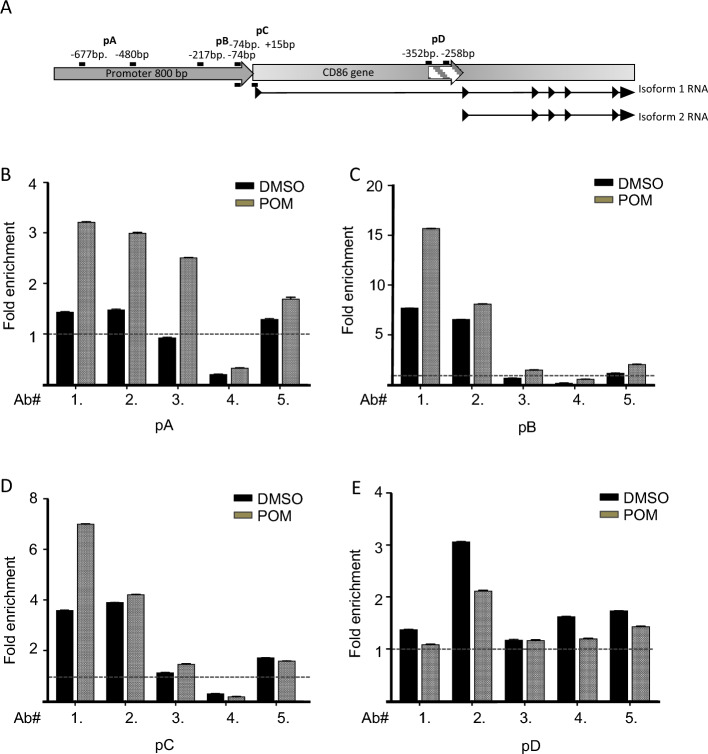
Pom increases PU.1 occupancy at the promoter of B7-2/CD-86. (**A**) Schematic for human B7-2 (CD86 gene) and promoter regions pA-pD. (**B**–**E**) Fold enrichment levels for PU.1 in DMSO and Pom (1 µM) treated WT Daudi cells at the four promoter regions pA-pD using 4 different PU.1 antibodies (ab 1–4) and one antibody to IRF8 (ab5). The fold enrichments are adjusted to the values obtained using the IgG control antibody. Dashed line shows the IgG value to which all the antibodies were normalized. Error bars represent the standard deviation for each lane and values are normalized to the actin mRNA level in the DMSO control for each protein extract. Promoter regions are pA (− 677 to − 480), pB (− 217 to − 74), pC (− 74 to + 15) and pD (− 325 to − 258) relative to each isoform’s transcription start site.

Previously, Kanada et al. showed that PU.1 binds to the B7.2/CD86 promoter and enhances the expression of isoforms 1 and 2 of CD86/B7-2 in BMDC cells^29^. The organization of the B7-2 promoter and its PU.1 binding regions pA – PD are shown in Fig. 6A. To determine if PU.1 plays a role in Pom-induced upregulation of B7-2 expression in Daudi cells, PU.1 occupancy on the promoter was assessed by chromatin immunoprecipitation assay (ChIP) in DMSO and Pom-treated Daudi cells. ChIP was performed with 4 antibodies (Ab1, Ab2, Ab3 and Ab4) specific to PU.1 and enrichment was determined by qPCR. We also tested an antibody to IRF8 in the ChIP (Ab5), as PU.1 is known to associate with this factor in certain cases^41^. We examined the primary putative B7-2 promoter region of isoform 1 and the putative promoter of isoform 2 (Fig. 6A), both of which contain PU.1 binding sequences (Fig. 6A). Four sets of primers were designed specifically to the regions that showed the highest accumulation of putative PU.1 binding sites in these respective promoters. Three sets of primers cover the primary promoter of isoform 1 of the B7-2 gene whereas one pair covers the internal promoter region of isoform 2 (designated pD) (Fig. 6A). As expected, and previously observed by other groups^29,30^, there was a small but consistent enrichment of PU.1 on the promoters of both B7-2 isoforms in the DMSO-treated cells as compared to IgG controls. This enrichment of PU.1 in the control samples supports a role of PU.1 in basal transcription (Fig. 6B,C,D,E). This basal binding of PU.1 on both promoters was very evident in case of Ab1 and Ab2 in regions pB and pC. In the primary promoter region pB, there was a 7.5 and 6.5-fold enrichment of PU.1 with Ab1 and Ab2, respectively (Fig. 6C). Ab4 failed to show significant PU.1 binding for pA,pB, and pC and the IRF8 antibody (Ab5) only showed modest increases over basal levels for the pA region (Fig. 6B). Importantly, upon Pom-treatment there was a significant enrichment over basal levels of PU.1 in regions of the primary promoter (pA, pB and pC) but not in the internal promoter of isoform 2 (pD) and this was particularly notable for the rabbit polyclonal ab1 (Fig. 6B,C,D). Upon Pom treatment, regions pB and pC of the B7-2 primary promoter showed a 15- and sevenfold enrichment, respectively, with Ab1 (Fig. 6C,D). Although PU.1 was enriched at the internal promoter of isoform 2 (pD (Fig. 6E), there was no increase in enrichment following Pom treatment suggesting that Pom-mediated enhancement of PU.1 binding is specific to the primary promoter regions of the B7-2 gene. We explored whether Pom-mediated specificity of PU.1 might be regulated, in part, by IRF8, which is known to physically interact with PU.1^41^ and is expressed in EBV-infected cell lines^42^. However, as assessed by Ab5 specific to IRF8, IRF8 was not substantially enriched at the primary or the internal promoters of B7-2 gene (See antibody 5 in Fig. 6B,C,D,E) suggesting PU.1 alone may be sufficient for enrichment following its phosphorylation. These results suggest that there is occupancy of PU.1 in the primary promoter region of the B7-2 gene in basal conditions of Daudi cells and that Pom treatment increases the enrichment of PU.1 by approximately twofold, consistent with a role of PU.1 in upregulating CD86/B7-2 transcription.

We had previously found that Pom increases surface expression of B7-2 in PEL cells, but this was not associated with an increase in its mRNA^14^. Consistent with this, while PU.1 was easily detectable on the promoter in Daudi cells it was essentially undetectable in BCBl-1 cells (Supplemental file 8). The lack of PU.1 in these cells may in part explain the failure of Pom to increase B7-2/CD86 expression^20^. We confirmed the lack of expression of PU.1 in BCBL-1 cells by analyzing nuclear and cytoplasmic extracts for the presence of PU.1 in the absence and presence of Pom. Consistent with previous studies, PU.1 was not detected in BCBL-1 cell extracts with or without Pom treatment but was readily detectable in nuclear CA46 cell extracts (a control B cell line) (Supplemental file 9A). Interestingly, CA46 is a EBV-negative Burkitt lymphoma line and like BL41-, Pom did not increase B7-2 expression in these cells (Supplemental file 9B) suggesting PU.1 alone is not sufficient for Pom to upregulate B7-2 but appears to require EBV infection as well.

### Pom increases B7-2 and ICAM-1 mRNA and surface expression in other EBV-infected cell lines

We then investigated whether Pom-induced increases in mRNA, protein and surface marker expression for B7-2 and ICAM-1 were similarly observed in other EBV-infected lines or virus-negative B cell lines. To explore this, we used EBV negative BL41 Burkitt lymphoma cells (BL41-), EBV-infected BL41 (BL41 +) cells^43^, and Namalwa EBV-positive Burkitt lymphoma cells that contain two integrated copies of the EBV genome^44^. Pom did not increase B7-2 or ICAM-1 mRNA in the uninfected BL41- cells but led to a 1.4-fold average increase in B7-2 mRNA expression in the EBV-infected BL41 + cells (Table 1). Unlike the case with Daudi cells, Pom also increased the expression of ICAM-1 mRNA by 1.35-fold in BL41 + cells. Similarly, Pom treatment of Namalwa cells increased expression of B7-2 and ICAM-1 mRNA 3.1 and 2.7-fold, respectively (Table 1).

**Table 1:** Effect of 1 µM Pom on expression of B7-2 and ICAM-1 mRNA in BL cell Lines.

Cell line	B7-2 (fold change)*				ICAM-1 (fold change)*			
	Expt. 1	Expt. 2	Expt. 3	**Avg**	Expt. 1	Expt. 2	Expt. 3	**Avg**
BL41-	0.8	0.4	0.3	0.6	0.7	0.2	1.0	0.5
BL4 +	1.2	1.9	ND	1.4	1.3	1.4	ND	1.4
Namalwa	2.0	4.8	2.5	3.1	3.2	2.1	1.4	2.7

*The values are the fold change in mRNA levels for B7-2 and ICAM-1 following 1 µM Pom treatment for 24 h over the DMSO control values and normalized to 18S RNA.

**Figure 7:**
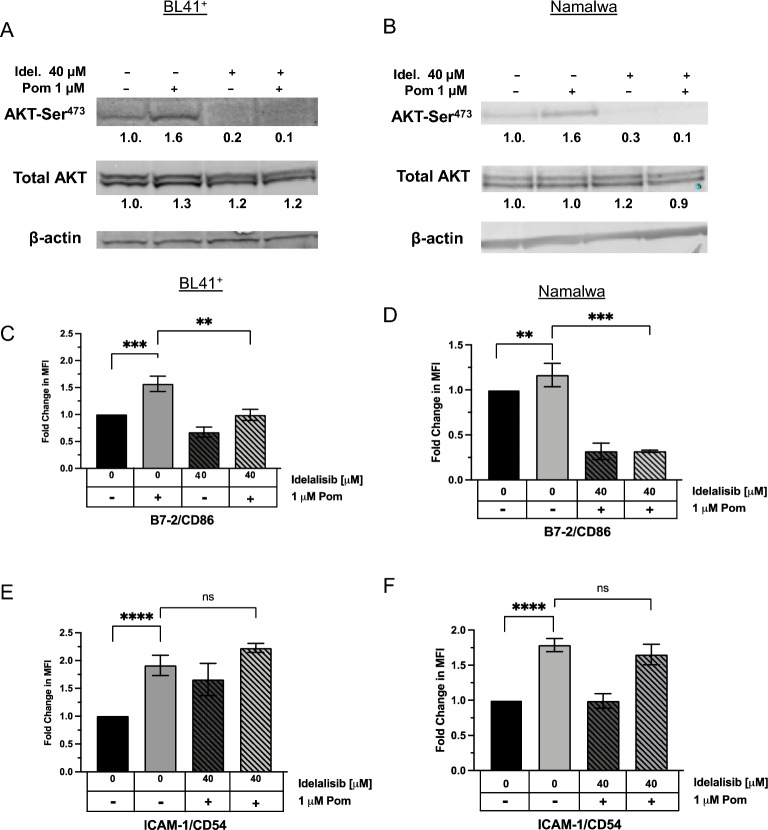
Idelalisib inhibits AKT-Ser^473^ phosphorylation and B7-2 surface expression induced by Pom in BL41 + and Namalwa cells. BL41 + and Namalwa cells were pretreated with DMSO or the PI3K δ-subunit inhibitor idelalisib for approximately 2 h and then treated with DMSO or 1 µM Pom for an additional 48 h. Representative immunoblots of cytoplasmic extracts show AKT phosphorylation levels with Pom treatment and/or idelalisib for (**A**) BL41 + cells or (**B**) Namalwa cells. Fold changes of AKT phosphorylation and total AKT are shown below the blot and calculated by first normalizing to β-actin. AKT phosphorylation was normalized to total AKT present after normalizing to actin. BL41 + and Namalwa cells treated as indicated in above were analyzed for surface expression levels of (**C**, **D**) B7-2 and (**E**, **F**) ICAM-1 by flow cytometry using PerCP/Cy5.5-conjugated anti-ICAM-1 or anti-B7-2 antibodies. Graphs show fold change in median fluorescent intensity (MFI) of surface expression levels measured via flow cytometry relative to DMSO-treated cells. The data are means + / − SD from 3 or more experiments and the statistically significant differences (****p ≤ 0.0001, ***p ≤ 0.001, **p ≤ 0.01, *p ≤ 0.05, ns not significant) between various treatments are indicated.

We next assessed whether the PI3K pathway is activated in these other EBV infected lines by measuring AKT-ser phosphorylation by immunoblot. AKT-ser phosphorylation was increased 1.6-fold by Pom treatment in BL4 + cells and in Namalwa cells, and basal and Pom-induced AKT phosphorylation was eliminated when pretreated with idelalisib (Fig. 7A,B). Similarly, B7-2 surface marker expression was increased by Pom 1.6 and 1.2-fold for BL41 + and Namalwa, respectively (Fig. 7C,D). Importantly, idelalisib inhibited the basal and Pom-induced expression of B7-2 (Fig. 7C,D). Cellular B7-2 expression was also increased by Pom in these two lines (Supplemental file 10). Idelalisib, however, was not able to suppress the Pom-induced increases in ICAM-1 suggesting again that the upregulation of ICAM-1 by Pom goes through a different pathway (Fig. 7E,F). Pom did not substantially increase B7-2 or ICAM-1 surface expression in the uninfected BL41- cells even at 5 or 10 µM Pom, or the cellular B7-2, suggesting that infection of the cells is required for the effects of Pom in BL lines (Supplemental file 10A,B). We also investigated the effects of Pom on the B-cell lymphoma line MC116. While Pom decreased the levels of B7-2 in uninfected MC116 cells, it increased its expression in the KSHV-infected line up to 1.7-fold (Supplemental file 10C). Taken together, these data suggest that Pom’s effects on B7-2 and ICAM-1 upregulation in certain lymphoma lines requires the presence of virus infection.

## Discussion

Our group has shown that treating KSHV-infected PEL or certain EBV-infected lymphoma B-cell lines with Pom leads to the upregulation of important immune surface markers including ICAM-1, B7-2, MICA, and MHC-I^19^. We have also shown that this treatment results in increased T-cell activation as well as increased natural killer cell activity toward these cell lines^15,19^. This activity may contribute to the therapeutic effects of Pom in these tumors, since an effective T-cell immune response against virus infection requires expression of MHC-I and/or co-stimulatory molecules such as B7-2 and ICAM-1 on target cells^45^. To explore the mechanism(s) for these effects, we have now developed cell lines resistant to Pom’s cytostatic effects by serially passaging these lines with increasing concentrations of Pom; these include a BCBL-1 Pom-resistant cell line^15^, and in this report, a Pom-resistant Daudi cell line. In both cases, resistance to Pom’s cytostatic effect is associated with a decrease in the expression of cereblon, the primary target of pomalidomide and related derivatives. Although Pom treatment of KSHV and EBV infected lines appear to require the presence of cereblon and ultimately lead to similar outcomes regarding increased immune surface markers and immune recognition, here we have found distinct differences in the mechanism leading to these effects in PEL lines versus EBV-associated tumor lines.

When we initiated these experiments, we hypothesized that Pom would upregulate immune surface markers in EBV-infected lines in the same way as in KSHV infected lines. Although Pom appears to require cereblon to activate the PI3K pathway in both KSHV and EBV-infected cell lines, the mechanism diverges from there. Most notable among the differences was that Pom induced increases in B7-2 mRNA in EBV infected cells while this was not observed in KSHV infected PEL cells. An underlying reason for this difference may be that KSHV and EBV-infected tumor lines differ in the expression of the important ETS transcription factor PU.1 which is important in myeloid and B-cell development. Here, we confirmed that BCBL-1 cells do not express PU.1, as reported by others^20^ and this is the case without or with Pom treatment. However, we found that Daudi cells and other non-Hodgkin EBV + lymphoma lines, including Namalwa, express significant levels of PU.1 consistent with previous reports^46^ and this may explain the higher basal expression of B7-2 compared to PEL lines. Since PU.1 has been shown to upregulate B7-2 in murine dendritic cells and more recently in human dendritic cells by binding directly to the promoter of B7-2^29,30^, we explored its potential role in Pom induced upregulation of B7-2 mRNA. Using ChIP assays, we demonstrated the presence of PU.1 at the promoter of B7-2 in WT Daudi cells and its absence at the promoter in control BCBL-1 cells. More importantly, we find that Pom treatment increased the levels of PU.1 in the nucleus and increased the levels of PU.1 associated with the B7-2 promoter in Daudi cells but not in BCBL-1 cells. It is important to note that PU.1 expression alone is not enough to lead to increased B7-2 following Pom treatment. CA46 cells express PU.1 but Pom does not increase the levels of PU.1 nor raise B7-2 in these cells. Thus, while PU.1 expression is required, it alone is not sufficient to induce B7-2 upregulation in EBV-negative BL lines.

We pursued the mechanism by which Pom might induce PU.1 activation in Pom treated Daudi cells. Our cytokine/chemokine data did not show increases in IFN-γ, IL-21, or IL-4, which have been shown to increase B7-2 in myeloma cell lines. However, we did consistently see increases in MIP-1∝/β and IP-10 after treatment of Daudi cells with Pom. We explored whether MIP-1∝ might be involved in the upregulation of ICAM-1 and B7-2 as MIP-1∝ has been shown to upregulate the PI3K pathway^28^, but we found no evidence for its involvement. However, when we further explored the PI3K pathway using an inhibitor of the delta subunit of PI3K, it was able to block the upregulation of B7-2 surface expression. Idelalisib blocked serine 473 phosphorylation of AKT in Daudi cells, which is required for phosphorylation of PU.1 by AKT^35^, and blocked the upregulation of B7-2 surface expression, but not that of ICAM-1. The PI3K/AKT pathway was only weakly activated by Pom treatment in the resistant Daudi cell line, suggesting that activation of that pathway is likely dependent on cereblon. Unlike the Daudi wild-type line, our Daudi cell line resistant to Pom showed an impaired ability to downregulate ikaros in response to Pom. Thus, Pom-induced degradation of ikaros by cereblon may lead to the activation of the PI3K pathway, as has been reported in leukemia cells^31^, and in the case of EBV-infected cells, this leads to an increase in PU.1 activation and upregulation of B7-2 mRNA.

A distinct difference between the mechanism of B7-2 upregulation in PEL and Daudi cells is the role played by the delta subunit of PI3K. Idelalisib, a specific inhibitor of the delta subunit of PI3K, was able to block AKT phosphorylation in Daudi cells and completely block the upregulation of B7-2 surface expression. By contrast, idelalisib used at the same and higher concentrations only minimally lowered the surface expression of B7-2 in PEL cells. Also, while ICAM-1 and B7-2 in PEL lines both seem to share a similar pathway that goes, in part, through PI3K^15^, there are differences in the mechanism for these two markers in EBV-infected cells. For example, inhibitors of the PI3K/AKT pathway had no inhibitory effect on the Pom-induced upregulation of ICAM-1 in Daudi cells. ICAM-1 expression has been shown to either be variably upregulated or downregulated by PI3K in multiple cell types indicating different mechanisms are present for this surface marker^47,48^.

We also explored the effects of Pom on ICAM-1 and B7-2 surface expression in two other EBV-infected BL lines (Namalwa and BL41 +) as well as the EBV negative BL counterpart, BL41-. Interestingly, although both BL41 − and BL41 + cells have downregulated CD54/ICAM-1 and CD86/B7-2 expression^49,50^, Pom only increased immune surface markers in the EBV positive BL41 line. Both Namalwa and BL41 + showed increases of B7-2 and ICAM-1 with Pom treatment although there were differences in the extent of upregulation among these EBV positive lines. We also studied the effects of Pom on the B-cell lymphoma line MC116 either uninfected or infected with the EBV-related virus, KSHV. Like that observed for the BL41 cells, only the infected MC116 cells showed responses to Pom. While our data demonstrate a clear difference between the effects of Pom on matched uninfected and infected lines (BL41 and MC116) it remains possible that certain non-virus infected lymphoma lines might also respond significantly to Pom with surface marker upregulation. For Daudi cells, we have previously demonstrated that the increases in B7-2, ICAM-1 and MHC class I polypeptide related sequence A (MICA) translate to increased immune recognition by natural killer cells and T-cells (Fig. 1 and^19^). The data presented here and reported previously suggests that Pom may be useful in the treatment of those BL associated with EBV infection.

It is also noteworthy that Pom treatment of Daudi cells significantly increased MIP-1∝/β and IP-10 levels in the supernatant of Pom-treated EBV-infected cells. MIP-1∝ and IP-10 levels have been associated with regressing BL tumors^51^. In fact, IP-10 was shown to mediate tumor necrosis of Burkitt lymphoma tumors^51^ and other antitumor responses in vivo^52^ suggesting that Pom could have additional effects on BL tumor regression in vivo due to the upregulation of IP-10. Additionally, therapy of tumors with individual chemokines including MIP-1∝ and IP-10 can induce tumor regression and immunity to subsequent tumor challenge (^53^ and references therein). Together these findings suggest that Pom may be useful in the treatment of EBV-infected lymphomas by two mechanisms. One, by enhancing immune recognition and the other by eliciting antitumor effects through the production of proinflammatory chemokines.

## Methods

### Cells and cell culture

The following cell types were used: Burkitt lymphoma B-cell lines Daudi and Namalwa (ATCC, Manassas, VA), EBV-negative BL41 (BL41 −) and EBV-positive BL41 (BL4 +) (a kind gift from Bill Sugden, UW, Madison), BCBL-1, JSC-1,^15^, PBMCs (IQ Biosciences), BC-2, HUVEC, and U937 monocytes (ATCC). Cell lines were grown in RPMI 1640 medium (Invitrogen, Carlsbad, CA) supplemented with 15% fetal bovine serum (FBS) (Thermo Scientific, Rockford, IL), 1% penicillin/streptomycin glutamine (Sigma, St. Louis, MO) at 37 °C with 5% CO_2_.

### Reagents

Pom was obtained from Celgene Corp. (now Bristol Myers Squibb) and was stored at -20ºC as 20 mM stocks in dimethyl sulfoxide (DMSO)(Sigma). Idelalisib, LY294002, (Selleck Chemicals, Houston, TX) were stored − 20 °C at 100 mM in DMSO. Cell viability during drug treatment was assessed by trypan blue staining. In all experiments, cells were treated with DMSO vehicle or 1 μM Pom for 48 h unless indicated otherwise.

### Generation and characterization of Pom-resistant Daudi cells

Daudi cells were cultured in 15% FBS/RPMI 1640 and 0.005 μM Pom and the Pom concentration was doubled upon each passage if viability was not impaired more than 30%. Once significant inhibition of growth was seen, the Pom concentration was maintained until cell growth recovered (significant inhibition of growth was seen between 0.04 µM and 0.08 µM Pom). Cells were ultimately made resistant to growth inhibition by Pom up to 8 μM. Pom-Resistant cells could be passaged without Pom for at least two months without showing loss of Pom resistance. For a control, Daudi cells were treated with equivalent amounts of DMSO and passaged an equal number of times as the Pom treated cells.

### T-cell activation

T-cell activation assays were performed using IL2-Jurkat T-cells (Promega, cat# J1651) as the effector cells according to the manufacturer’s recommended protocol and as described previously ^15^. Briefly, 3 × 10^5^ Daudi cells per mL were treated with DMSO control or indicated concentrations of Pom for 2 days. They were then co-incubated with 10^5^ IL2-Jurkat T-cells at a 1:5 target to effector ratios in a 37 °C incubator and stimulated with 10 µg/mL of anti-human CD3 monoclonal antibody (OKT3 from ThermoFisher Scientific, cat# 16–0037-81). Relative light units (RLU) were measured after 6 h using Victor X3 multilabel plate reader (PerkinElmer), and background luminescence from media control was subtracted from all wells. Fold change in activation by Pom was determined after subtracting baseline RLU obtained from Jurkat cells incubated alone from that obtained from Jurkat cells co-incubated with Daudi cells.

### Flow cytometry analysis

Analysis of live (unfixed) cells for surface marker expression was carried out as described previously^14^. PerCP-Cy5.5-labeled isotype or antibodies toward CD86 (cat # 374,215), and CD54 (cat # 353,119) were purchased from Bio-legend, San Diego CA. Analysis was conducted using a flow cytometry Calibur™ Flow Cytometry system (BD Biosciences, San Jose, CA) followed by FlowJo flow cytometry analysis software (flowjo.com).

### Immunoblotting

Nuclear and cytoplasmic extracts were prepared from 2 × 10^6^–4 × 10^6^ live cells using the NE-PER Nuclear Extraction Reagent kit (ThermoFisher Scientific, Waltham, MA) with Halt Protease Inhibitors Cocktail (Pierce, Rockland, IL) and 1 mM ethylenediaminetetraacetic acid (EDTA). Lysates used for the analyses of phosphorylated AKT were extracted in the presence of both phosphatase inhibitor cocktail (Cell signaling cat#5870) and protease inhibitor cocktail (ThermoFisher Scientific) at a final 1X concentration. Protein concentrations were determined using the BCA assay (ThermoFisher Scientific). Samples of equal protein content were subjected to LDS-PAGE (4 to 12% NuPAGE Tris-Bis) (Invitrogen, Carlsbad, CA) and transferred to nitrocellulose membranes using iBlot (Life Technologies Grand Island, NY). Membranes were then blocked with Odyssey blocking buffer (Li-Cor, Lincoln, NE) for 1 h. Blots were incubated overnight with indicated antibodies: rabbit PU.1 antibody (PA5-17,505), ThermoFisher Scientific), mouse anti-β-actin (Sigma, cat# A2228), mouse anti-ICAM-1 (Abcam), mouse anti-B7-2 (R&D systems) mouse anti-IKZF1 (Santa Cruz, cat# 398,265), mouse anti-Cereblon, (Sigma). Primary antibodies against AKT were rabbit anti-phospho (Ser473)-AKT (cat# 4060) and mouse anti-AKT-pan (cat# 2920) (Cell Signaling) subjected to the appropriate secondary antibodies conjugated to green or red fluorescent dyes for 1 h (Li-Cor). Blots were washed three times using 10% TBST. Quantitation of protein on immunoblots was determined using Image Studio software (Li-Cor). The fold-change values for protein bands shown below the immunoblots represent the intensity of the test protein normalized to that the intensity of the control proteins (actin or TB). The control lane is set to 1 and all test values normalized to the control.

### Cytokine/Chemokine analysis

Cells were treated with DMSO as the control or Pom and incubated for 48 h. The supernatants from the cells were collected following centrifugation of the cells at 500G for 10 min. The supernatant (0.5 ml) was then used to analyze the relative levels of 36 different cytokines/chemokines using the Proteome Profiler Human Cytokine Array Kit (R&D, Catalog # ARY005B). After removal of the supernatant from the exposed membranes, they were then incubated with a fluorescent labeled streptavidin antibody at 1:2000 (Li-Cor, #926–32,230), washed and scanned. Image-Studio was used to quantify the intensities of the spots on the membrane, in duplicate, and the average was taken and used as the level of intensity.

### Real-time quantitative reverse transcription PCR

After treatment, mRNA was extracted using Qiagen™ kit (ThermoFisher Scientific, Waltham MA). cDNA synthesis was performed using High-Capacity cDNA Reverse Transcription kit (ThermoFisher Scientific) on a T100 Thermal Cycler (Bio-Rad). q-PCR reaction setup using SYBR-green included enzyme activation at 95 °C for 10 min and 40 cycles of amplification at 95 °C for 15 s and 60 °C for 1 min followed by melting curve analysis. Expression was normalized to 18S endogenous control RNA and quantification of relative mRNA expression was performed using ∆∆Cт method. The following primers (5’ to 3’) were used in for q-PCR:

18S: GCCCGAAGCGTTTACTTTGA and TCCATTATTCCTAGCTGCGGTATC, Primers for B7-2 and ICAM-1 were from Bio-Rad (B7-2: 10,025,636, qHsaCED0043530) and (ICAM-1: 10,025,636, qHsaCED0004281) (Hercules, CA).

### Chromatin immunoprecipitation (ChIP) assay

Chromatin immunoprecipitation (ChIP) assays were performed using an Abcam ChIP kit (Ab500) according to the manufacturer’s instructions. Briefly, Daudi cells were treated with 1 µm Pomalidomide or equivalent amount of carrier DMSO. Treatment was performed 2 h post seeding of 0.2 million per mL Daudi cells. Twenty hours post incubation, 5 million cells were collected and washed with PBS. Cells were fixed with 1.1% of formaldehyde for 10 min at RT and samples were neutralized using glycine followed by lysis in Buffer C. Lysis reaction was stopped by addition of 100 µl buffer D followed by sonication for 10 min (cycle: 25 s on/off) in a Diagenode Bioruptor. For the ChIP, 10 µg of four different PU.1 antibodies; Ab#1 (PA5-115,807, Invitrogen), Ab#2 (6688-MSM2, Neo Biotechnologies), Ab#3 (PA351558, Invitrogen), Ab#4 (ab227835, Abcam), Ab#5 (PA17505, Invitrogen), an IRF8 antibody (D20D8, Cell Signaling technology) and rabbit IgG (Sigma) were incubated overnight with the sheared chromatin. Antibody pull-down was performed with Protein-A beads (Thermo) and bound beads were washed 5 times in wash buffer to remove non-specifically bound material. Bound DNA was isolated using a DNA purifying bead slurry provided with the kit. The amount of chromosomal DNA immunoprecipitated by each antibody was determined by quantitative PCR using the primers indicated (Supplemental File 1) and qPCR was performed using QuantStudio 3 (Applied Biosystems). Fold enrichment method was used to calculate the abundance of each fragment for different antibodies.

### Statistical analysis

Statistical analysis was performed using two-tailed student’s *t* test (paired) on experiments with 3 or more biological replicates. *P* values less or equal to 0.05 were considered statistically significant. Asterisks indicate *p* values: *p < 0.05, **p < 0.01, ***p < 0.001, ****p < 0.0001.

## Supplementary Information

Supplementary Information.

## Data Availability

The data that support the findings of this study are presented in the main text or the supplementary material. Additional data that support the findings of this study are available from the author, [R.Y.], upon reasonable request and/or will be deposited in the Dryad repository.

## Abbreviations

DMSO: Dimethyl sulfoxide
FACS: Fluorescent activated cell sorter
Pom: Pomalidomide
HHV-8: Human HerpesVirus-8
EBV: Epstein Barr Virus (HHV5)
KSHV: Kaposi sarcoma associated herpesvirus (HHV8)
KS: Kaposi sarcoma
IKZF1: Ikaros Family Zinc Finger 1
PI3K: Phosphoinositide 3-kinase
