# Supplementary material for: Mechanism and therapeutic implications of pomalidomide-induced immune surface marker upregulation in EBV-positive lymphomas
Source: Sci Rep. 2023 Jul 18;13:11596. doi: 10.1038/s41598-023-38156-z (PMC10354044; doi:10.1038/s41598-023-38156-z)
Supplement: Supplementary file 1 — Supplementary Information. [file 41598_2023_38156_MOESM1_ESM.pdf]

Mechanism and Therapeutic Implications of Pomalidomide-Induced Immune Surface Marker Upregulation in EBV-Positive Lymphomas

Jaeger and Davis et al.

Supplemental Files

Supplemental file 1

Primer name	Sequence	Isoform
pA-677	AAGTTTCTAGATGATGCCC	Isoform 1
pA-480	GAGGGCAGACAGAAGCTACT	Isoform 1
pB-217	GAGGGAATTTCAAGAGGGGAGA	Isoform 1
pB_2-74	ACAGATTTTGACCACACTTGAGG	Isoform 1
pC-74	CCTCAAGTGTGGTCAAAATCTGT	Isoform 1
pC-15	TCCTCGGCAATGACTGTATACC	Isoform 1
pD-352	GGCCCTTGGACATTTCCTCT	Isoform 2
pD-258	GCCAATGGAATTGTGGGCAG	Isoform 2

Supplemental file 2

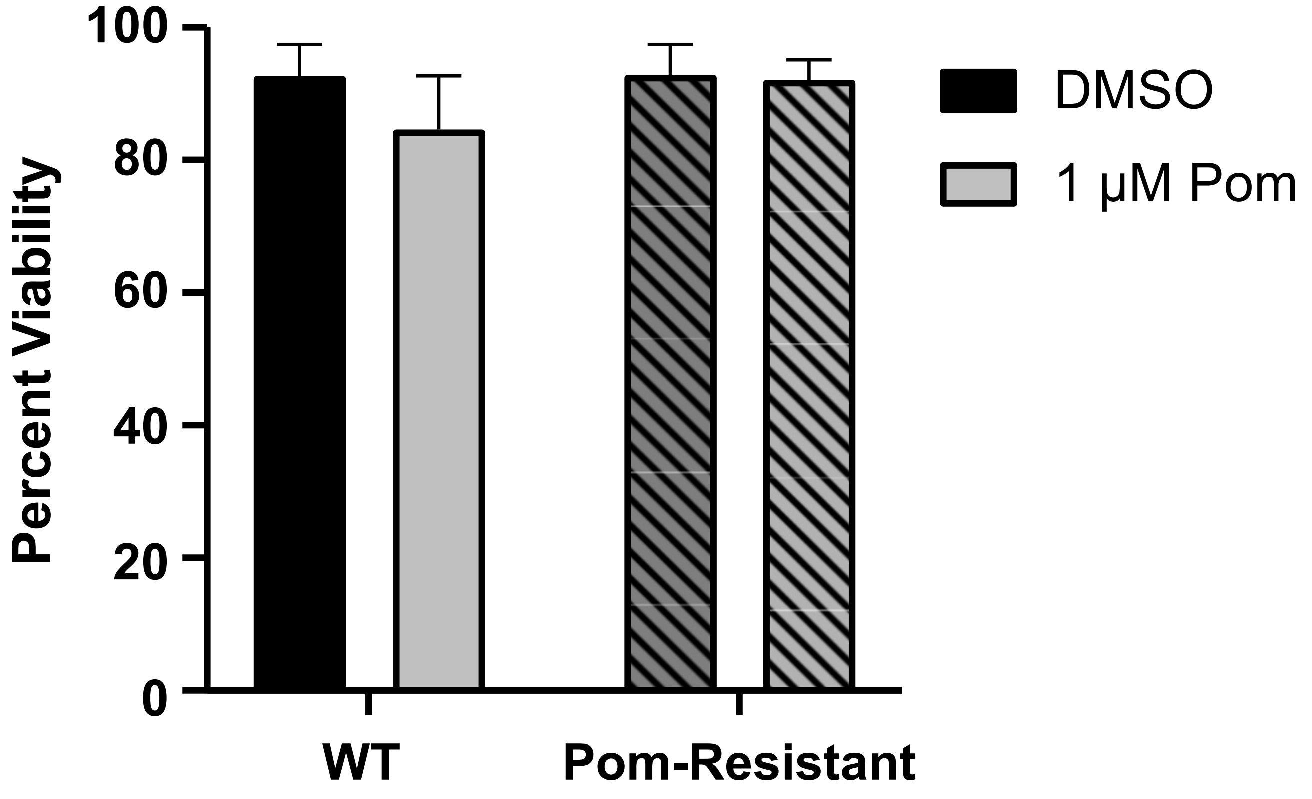

Supplemental file 3

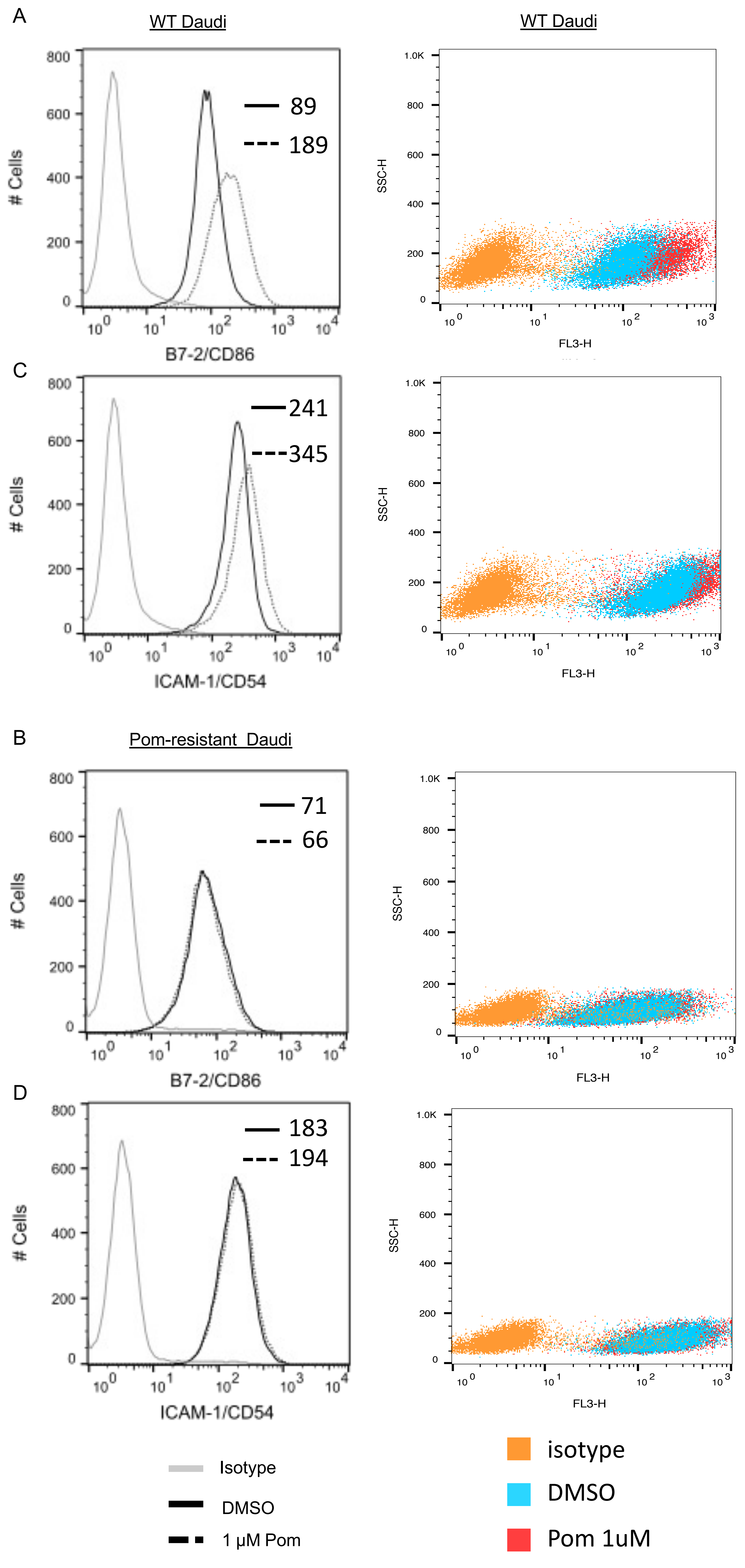

Supplemental file 4

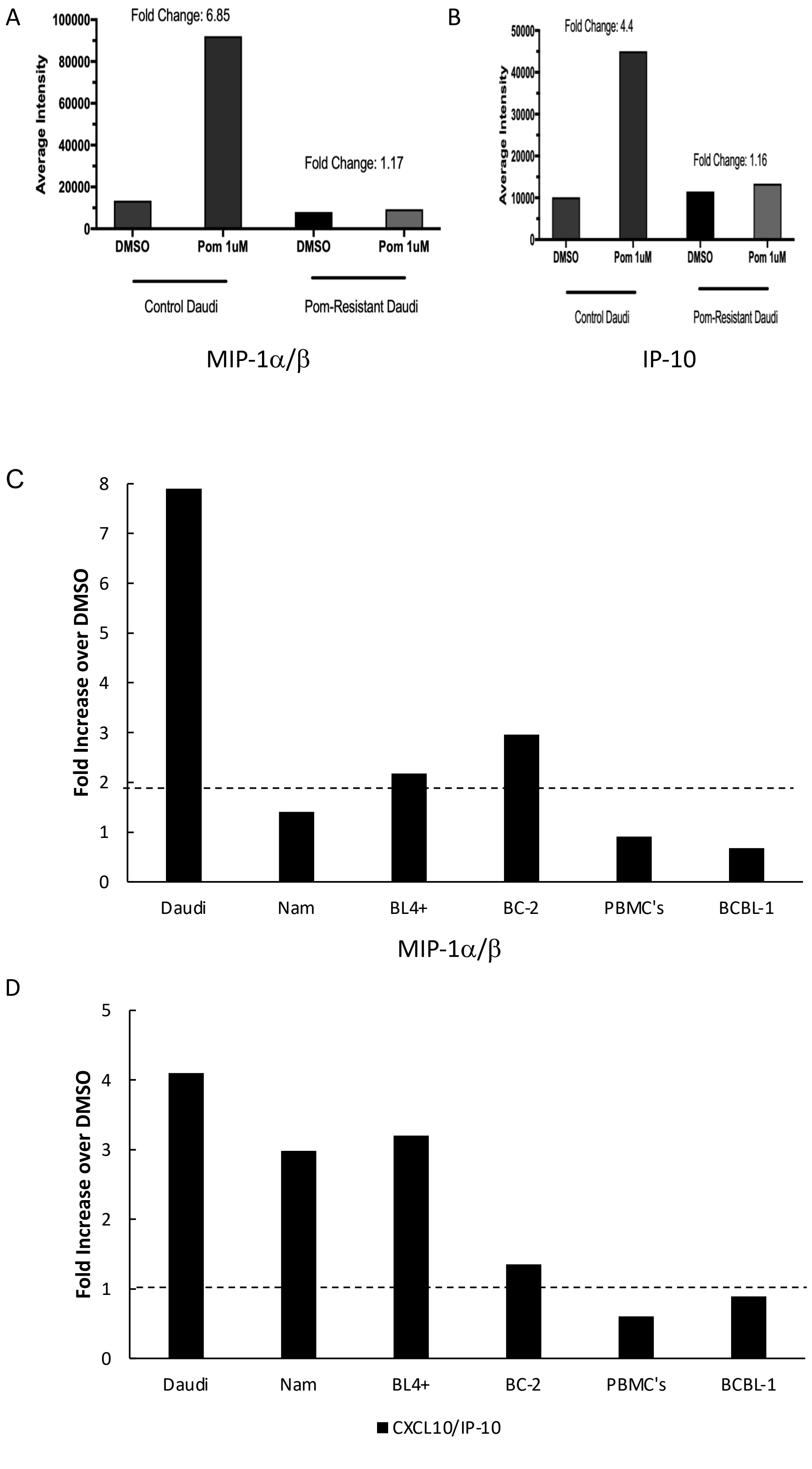

Supplemental file 5

Daudi Control

B7-2

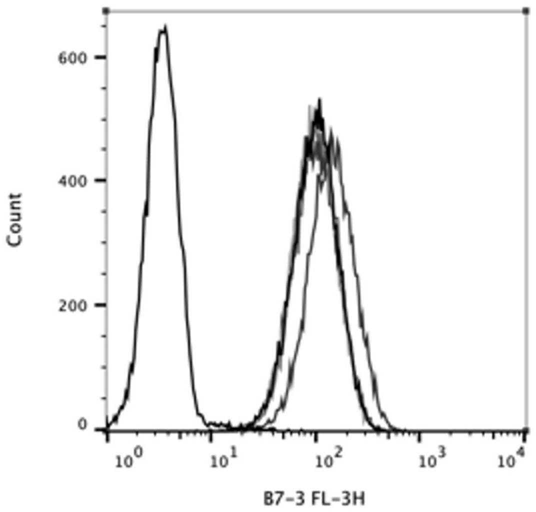

	Sample Name	Workspace Annotation	Subset Name	Median : FL3-H
☐	WT.001	isotype CRTL	WT	3.43
☐	WT.006	B7-2 CTRL	WT	98.7
☐	WT.007	B7-2 Pom 1uM	WT	139
☐	WT.008	B7-2 10nM MIP1-alpha	WT	95.8
☐	WT.009	B7-2 100nM MIP1-alpha	WT	99.8
☐	WT.010	B7-2 500nM MIP1-alpha	WT	96.7

Daudi Pom-Resistant

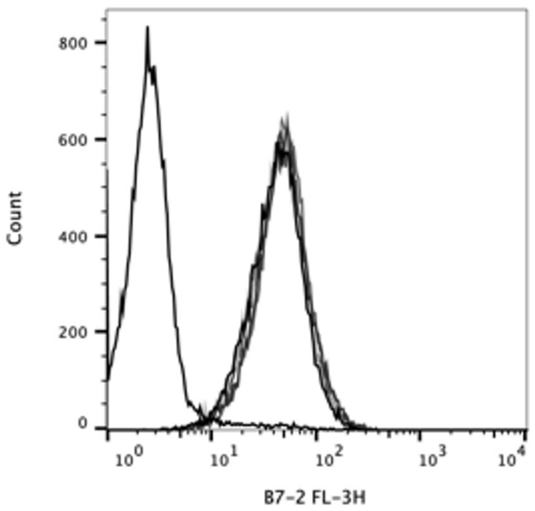

	Sample Name	Workspace Annotation	Subset Name	Median : FL3-H
☐	POMR.002	isotype	PomR	2.57
☐	POMR.006	B7-2 CTRL	PomR	42.5
☐	POMR.007	B7-2 Pom 1uM	PomR	47.5
☐	POMR.008	B7-2 10nM MIP1-alpha	PomR	43.6
☐	POMR.009	B7-2 100nM MIP1-alpha	PomR	48.6
☐	POMR.010	B7-2 500nM MIP1-alpha	PomR	44.2

ICAM-1

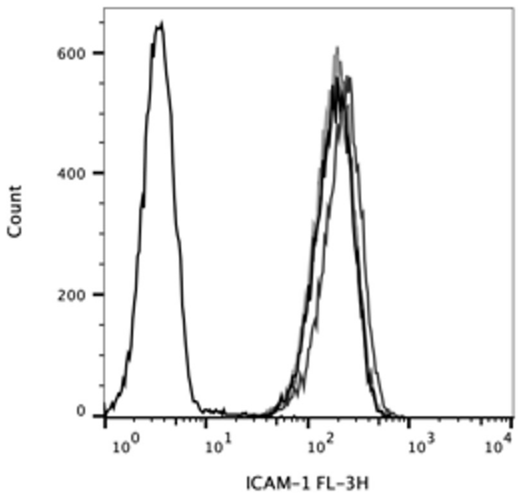

	Sample Name	Workspace Annotation	Subset Name	Median : FL3-H
☐	WT.001	isotype CRTL	WT	3.43
☐	WT.011	ICAM-1 CTRL	WT	179
☐	WT.012	ICAM-1 Pom 1uM	WT	218
☐	WT.013	ICAM-1 10nM MIP1-alpha	WT	177
☐	WT.014	ICAM-1 100nM MIP1-alpha	WT	181
☐	WT.015	ICAM-1 500nM MIP1-alpha	WT	181

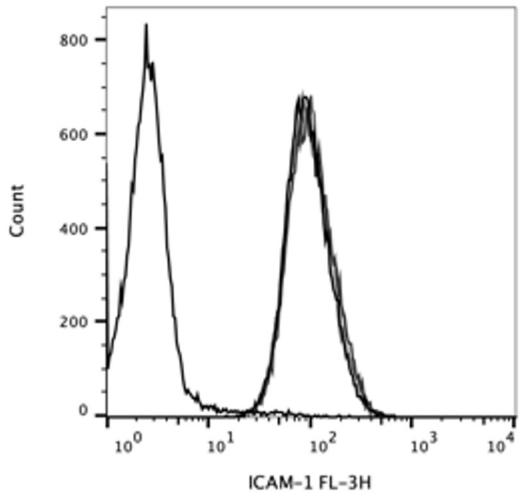

	Sample Name	Workspace Annotation	Subset Name	Median : FL3-H
☐	POMR.002	isotype	PomR	2.57
☐	POMR.011	ICAM-1 CTRL	PomR	90.6
☐	POMR.012	ICAM-1 Pom 1uM	PomR	96.5
☐	POMR.013	ICAM-1 10nM MIP1-alpha	PomR	92.2
☐	POMR.014	ICAM-1 100nM MIP1-alpha	PomR	90.2
☐	POMR.015	ICAM-1 500nM MIP1-alpha	PomR	96.0

Supplemental file 6

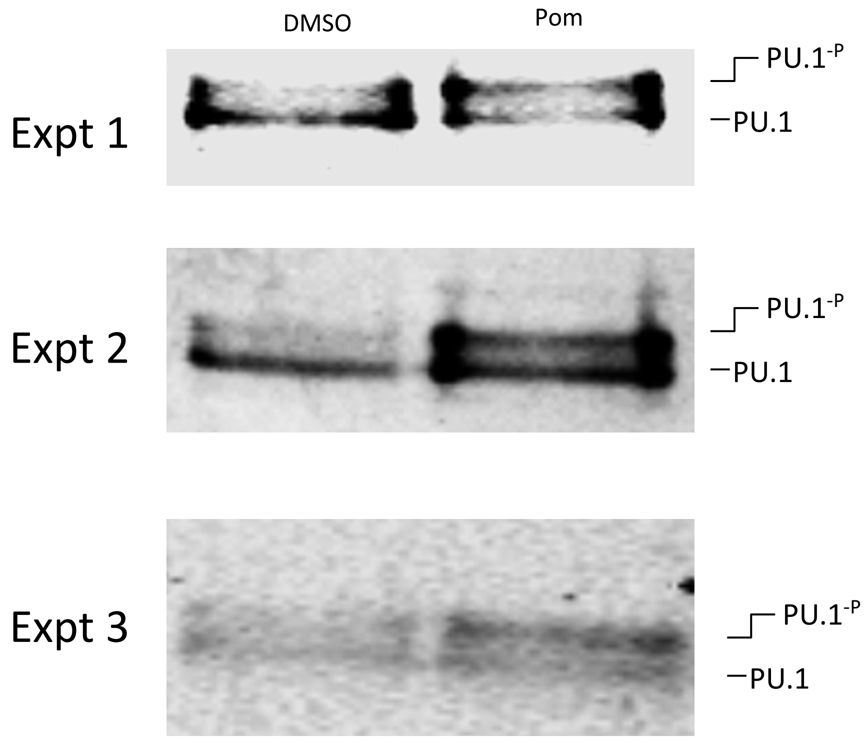

Supplemental file 7

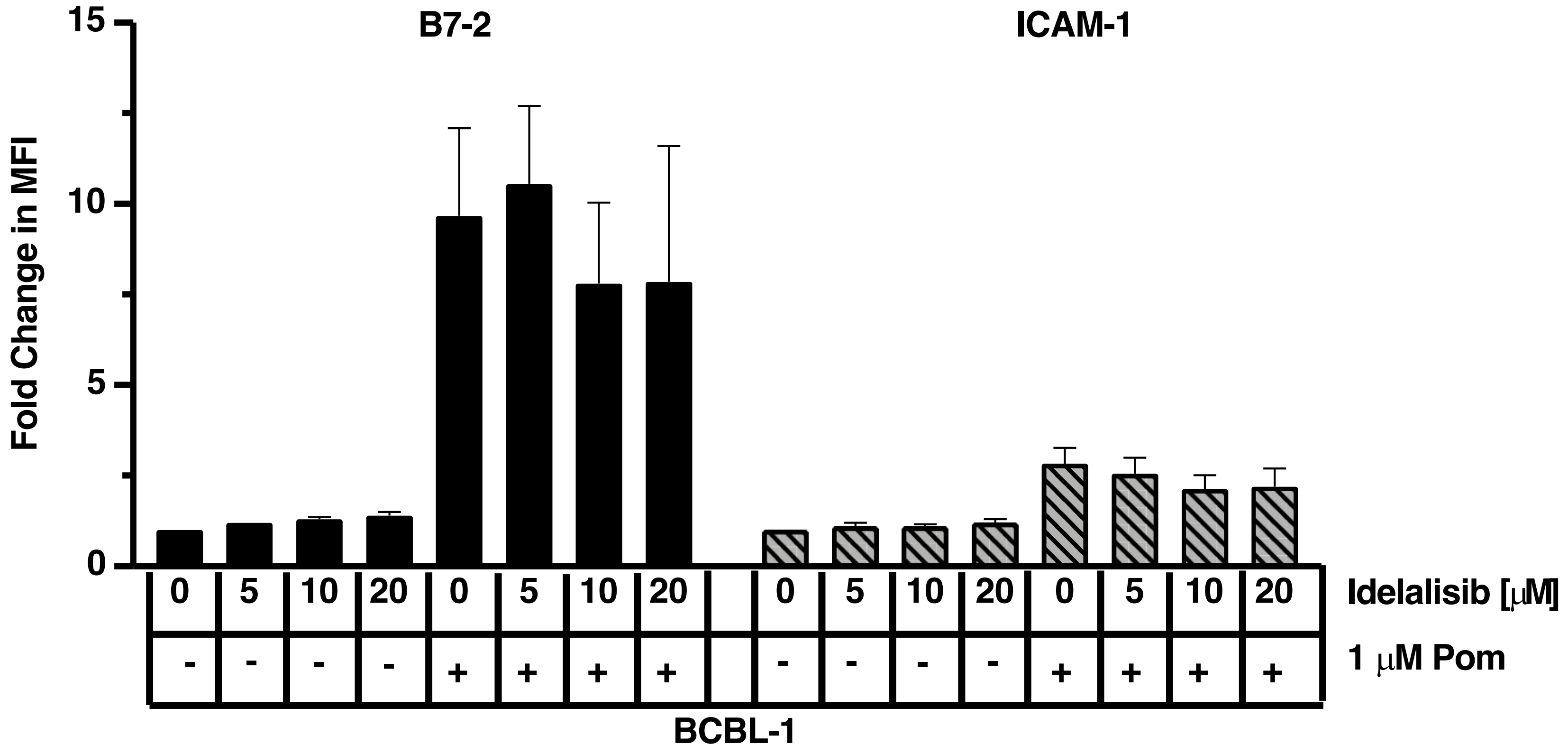

Supplemental file 8

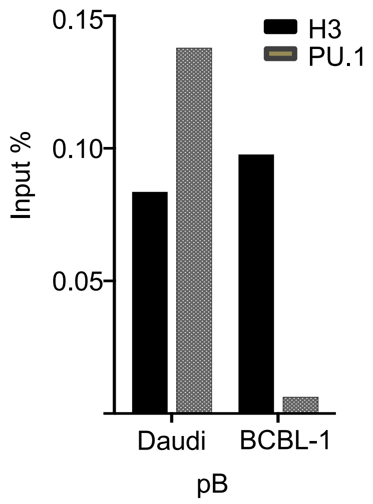

Supplemental file 9

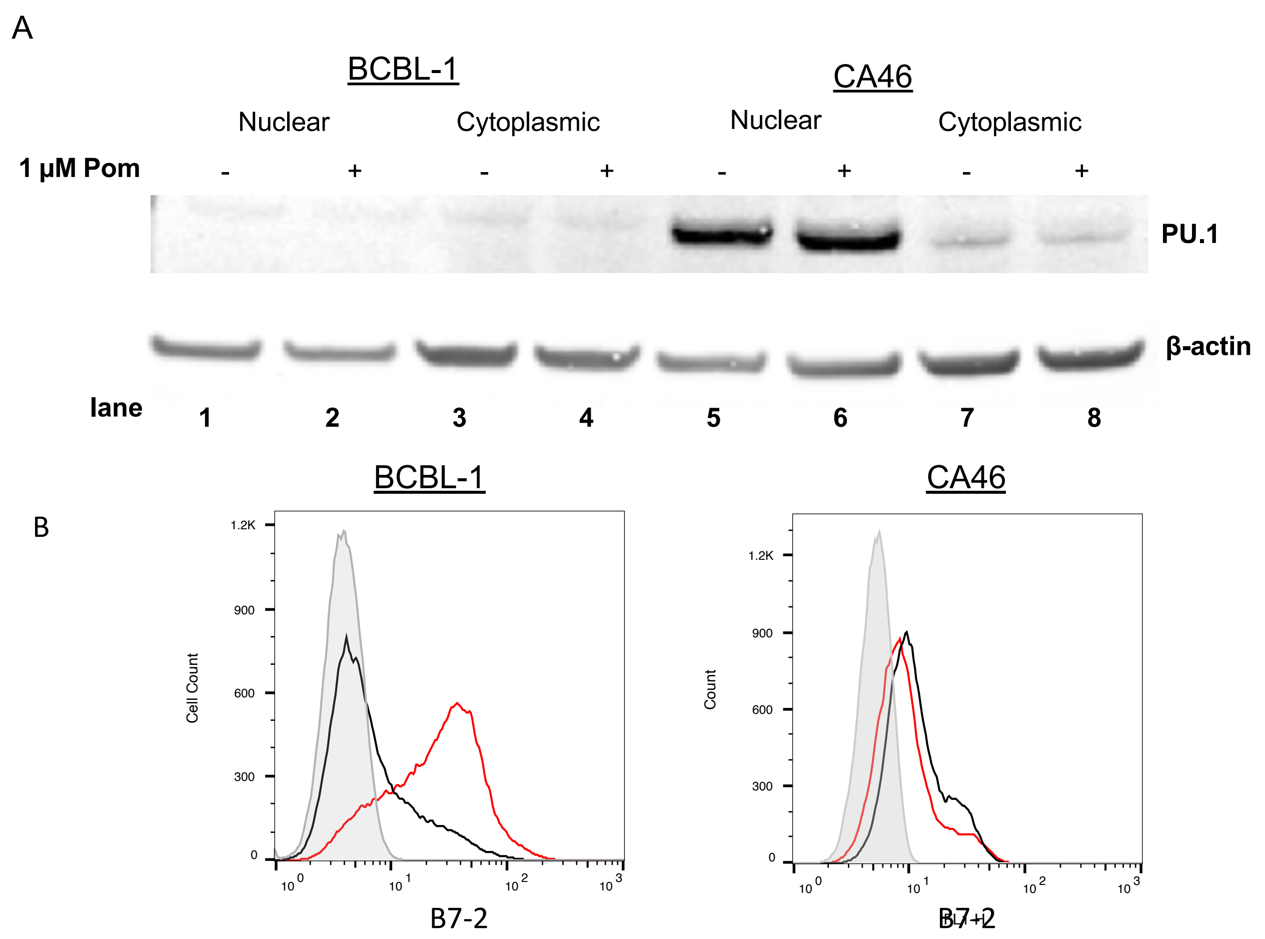

Supplemental file 10

A

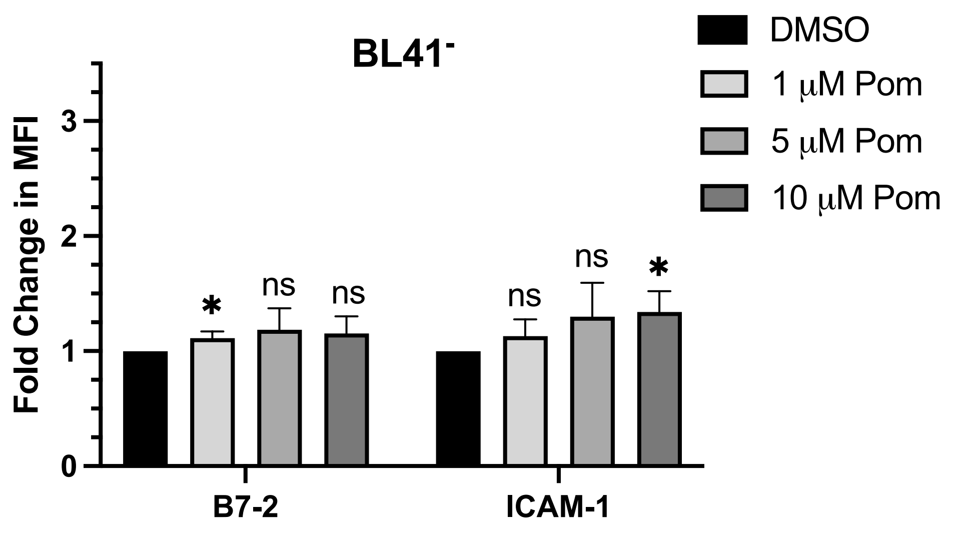

B

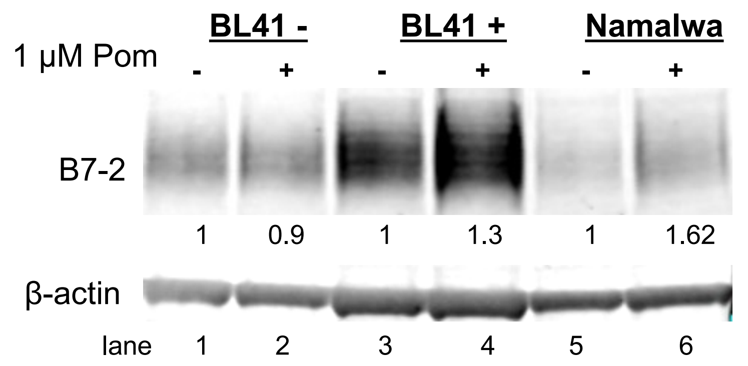

C

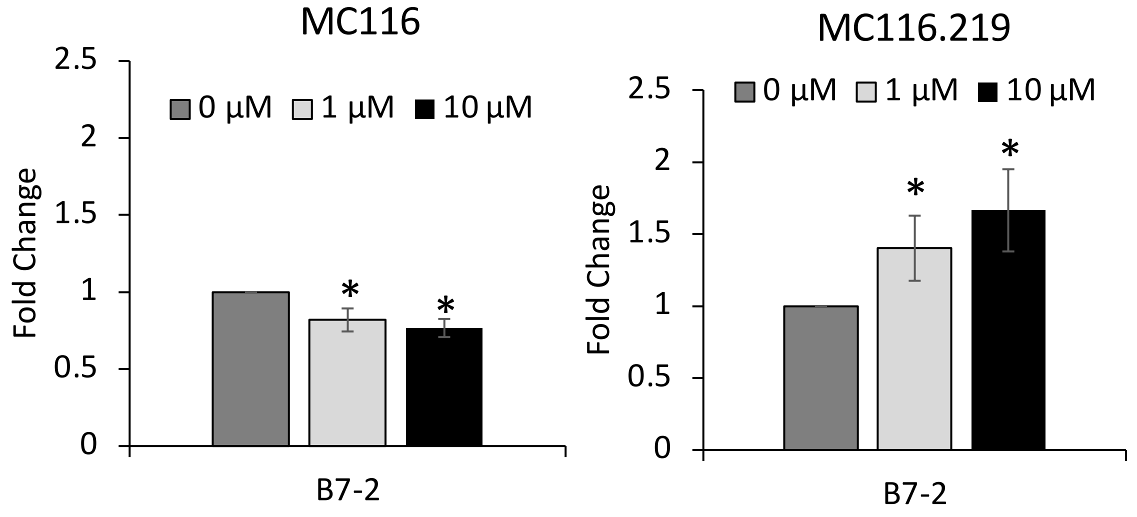

Supplemental file 11

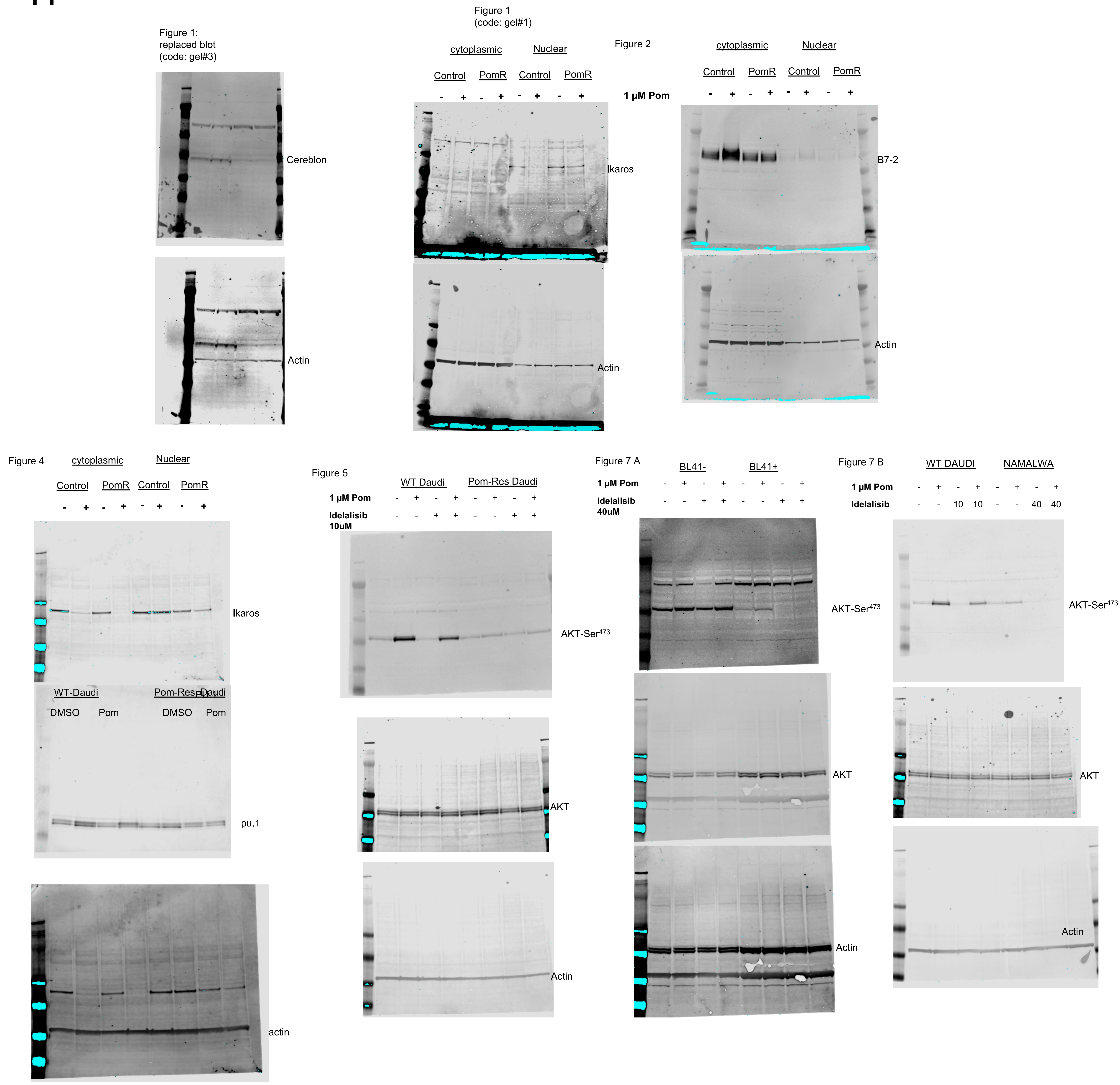

Mechanism and Therapeutic Implications of Pomalidomide-Induced Immune Surface Marker Upregulation in EBV-Positive Lymphomas

Jaeger and Davis et al.

Supplemental Figure Legends

Supplemental file 1: Primers used in the quantitative PCR analysis of the B7-2/CD86 human promoter. Shown are the primers that were used for the different isoforms for B7-2/CD86 in QT-PCR for the ChIP assays.

Supplemental file 2: **Pom has little effect on the viability of Control and Pom-resistant Daudi cells**. Control and Pom-resistant cells were cultured in the absence or presence of 1 µM Pom. After 48 hours live/dead analysis was performed using trypan blue staining. Percent viability (% of live cells over total) were calculated and presented as % of DMSO-treated cells. Data is presented as an average from 13 experiments for WT Daudi (sold bars) and 10 experiments for Pom-Resistant Daudi cells (hashed bars).

Supplemental file 3: Daudi cells made resistant to Pom induced cytotoxicity no longer upregulate B7-2 and ICAM-1 following Pom treatment. A, B) Surface expression for B7-2/CD86 in (A) WT or (B) Pom-resistant Daudi cells 48hr after treatment with DMSO (solid) or 1µM Pom (dashed) (C, D) Surface expression for ICAM-1 in (C) WT or (D) Pom-resistant Daudi cells 48hr after treatment with DMSO (solid) or 1µM Pom (dashed). Isotype controls shown in grey. The median fluorescent values are shown for the FACS profiles for clarity, the media fluorescent

value for isotype was 3.0. Shown to the right are the corresponding scatter plots from the FACS analysis example, 1 of 7 experiments.

Supplemental file 4: Pom increases MIP-1α/β and IP-10 in EBV-infected cells but not in Pom-resistant Daudi cells or other cell lines. (A) Relative MIP-1 α/β and (B) IP-10 levels in the supernatant of WT or Pom-resistant Daudi cells treated with 1 μM Pom for 24 hrs. (C) Relative MIP-1 α/β levels or (D) IP-10 levels performed in duplicate in the supernatant of Daudi, Namalwa, BL4+, BC-2, PBMC's or BCBL-1 cells treated with 1 μM Pom for 24 hrs. Samples were analyzed for changes in cytokine expression using the Proteome Profiler Human Cytokine Array Kit.

Supplemental file 5: MIP-1α does not increase the surface expression of B7-2 and ICAM-1 in Daudi cells. WT or Pom-resistant Daudi cells were treated with 1 μM Pom or the indicated concentrations of MIP-1α (10, 100 and 500 nm) for 48 hrs. Then cells were analyzed for B7-2 and ICAM-1 expression. Pom treatment was used as a positive control.

Supplemental file 6: Pom increases the levels of PU.1 as determined by immunoblot. (A) Protein levels by immunoblot using MOPS buffer (InVitrogen) of PU.1 running as a doublet from three different experiments. Pom induced increases in overall PU.1 as well as increased upper band consistent with a phosphorylated form.

Supplemental file 7: Idelalisib, an inhibitor of PI3K δ-subunit, does not block B7-2 and ICAM-1 upregulation in PEL cells. BCBL-1cells were pretreated with idelalisib for approximately 1 hour and then treated with 1 μM Pom for an additional 48 hrs. Surface expression levels of B7-2 and ICAM-1 were then measured by flow cytometry using PerCP/Cy5.5-conjugated anti- ICAM-1 or

anti-B7-2 antibodies in cells treated with Pom (1 µM) and/or idelalisib (at indicated concentrations). Graph shows fold changes in median fluorescence intensity (MFI) of B7-2 (black bars) and ICAM-1 (hashed bars) in BCBL-1 upon treatment with Pom and/or idelalisib relative to DMSO-treated cells. Shown are the means ± standard error for 2 separate experiments.

Supplemental file 8: PU.1 is easily detected at the promoter of B7-2/CD-86 in Daudi cells but not in BCBL-1 cells. H3 and PU.1 as a percentage of the input for Daudi cells or BCBL-1 cells. The H3 antibody (Abcam) and PU.1 polyclonal antibody (PA17505, Invitrogen) was used in the pulldown. Values are reported as a percent of the input.

Supplemental file 9: PU.1 is not detected in BCBL-1 cells without or with Pom treatment. A. Cells were treated with DMSO or 1 µM Pom for 24 hrs and then nuclear and cytoplasmic extracts were analyzed by immunoblot for PU.1 and actin. B. BCBL-1 cells (left panel) or CA-46 (right panel) were treated with Pom (1 µM) for 48 hrs then harvested and stained for B7-2 expression. Grey is isotype control, DMSO (solid black tracing) and Pom (solid red tracing).

Supplemental file 10: Pom does not upregulate B7-2 surface expression or protein expression in BL41- cells or in uninfected MC116 cells. (A) Fold changes in median fluorescence intensity (MFI) for Surface expression levels in BL41- cells for B7-2 and ICAM-1 measured by flow cytometry. Shown are the means ± standard deviations of at least 3 separate experiments. The statistically significant differences (*$p \leq 0.05$, ns not significant) between control and Pom treatments are indicated. (B) BL41-, BL41+ and Namalwa cells were treated with Pom for 48 hrs and then cytoplasmic extracts were prepared and analyzed by immunoblot. Fold changes shown under each lane are values normalized to the actin level in the DMSO control for each.

(C). MC116 cells uninfected or KSHV-infected ($2x10^5$ cells/mL) were treated with DMSO control (0µM), 1 µM, or 10µM Pom for 48 hours and then surface levels of B7-2 was measured by flow cytometry. Fold changes in median fluorescence intensity (MFI) for B7-2 relative to DMSO control are shown.

Supplemental file 11: Full versions of all western blots used in Figures. These full versions are the acquired TIFF files from the LiCor scanning using Image studio. These files have not been adjusted in any way.
